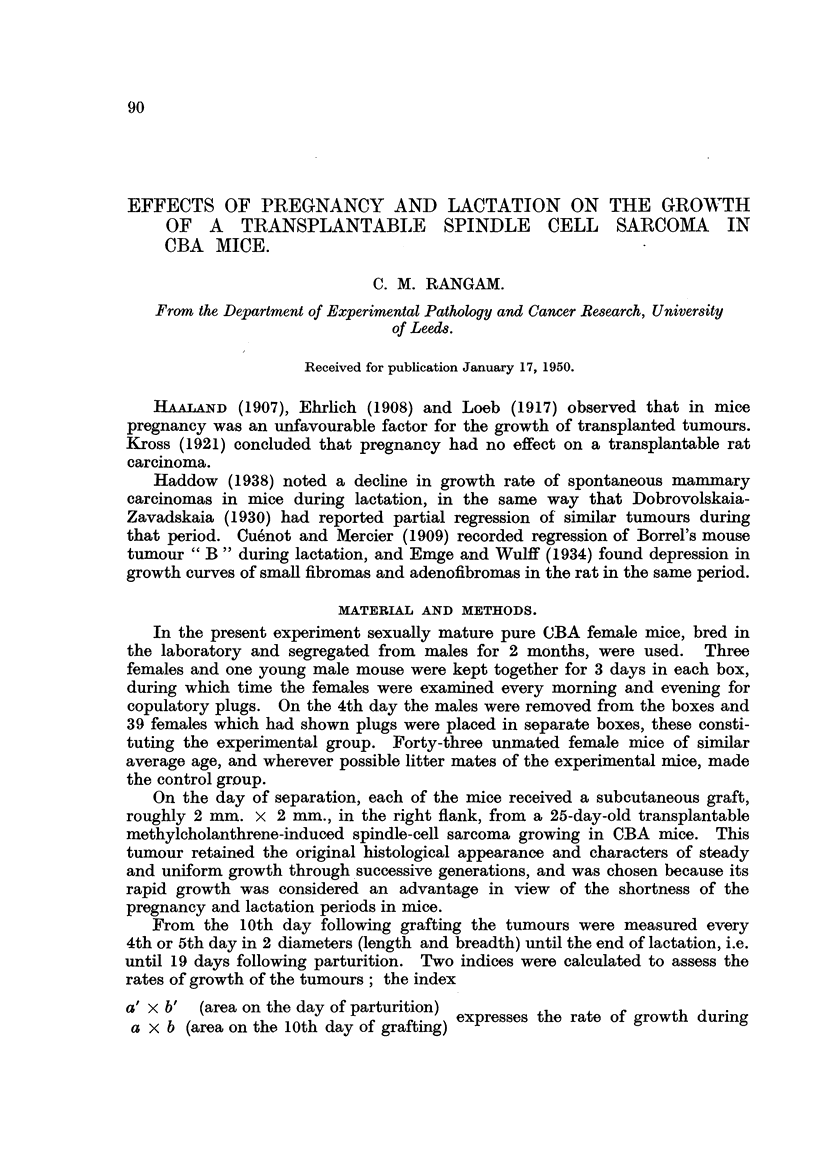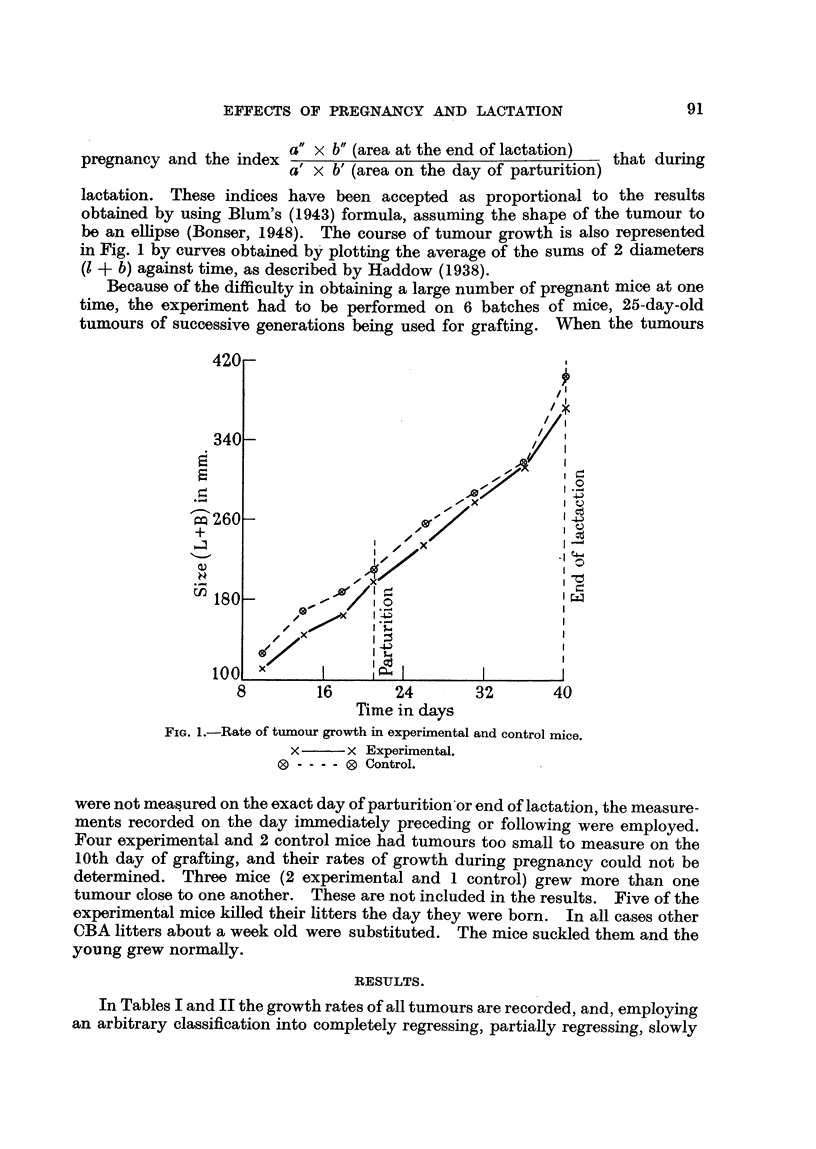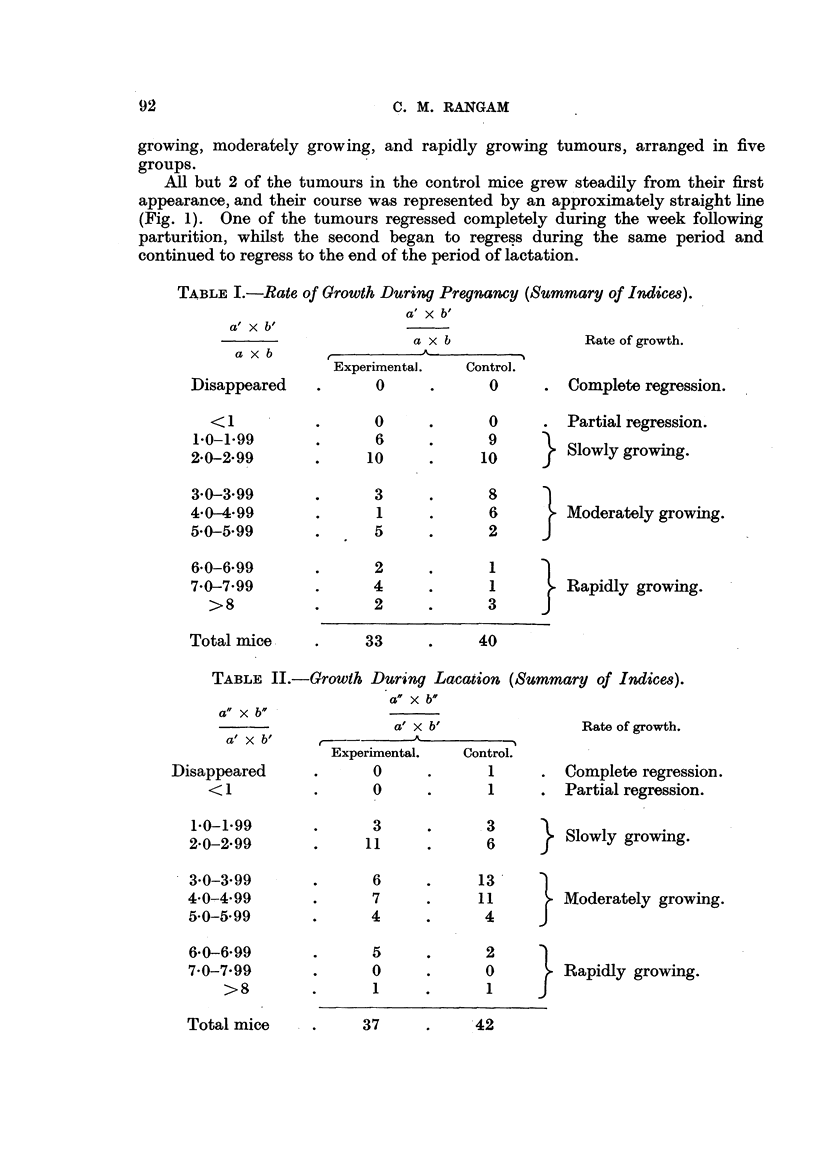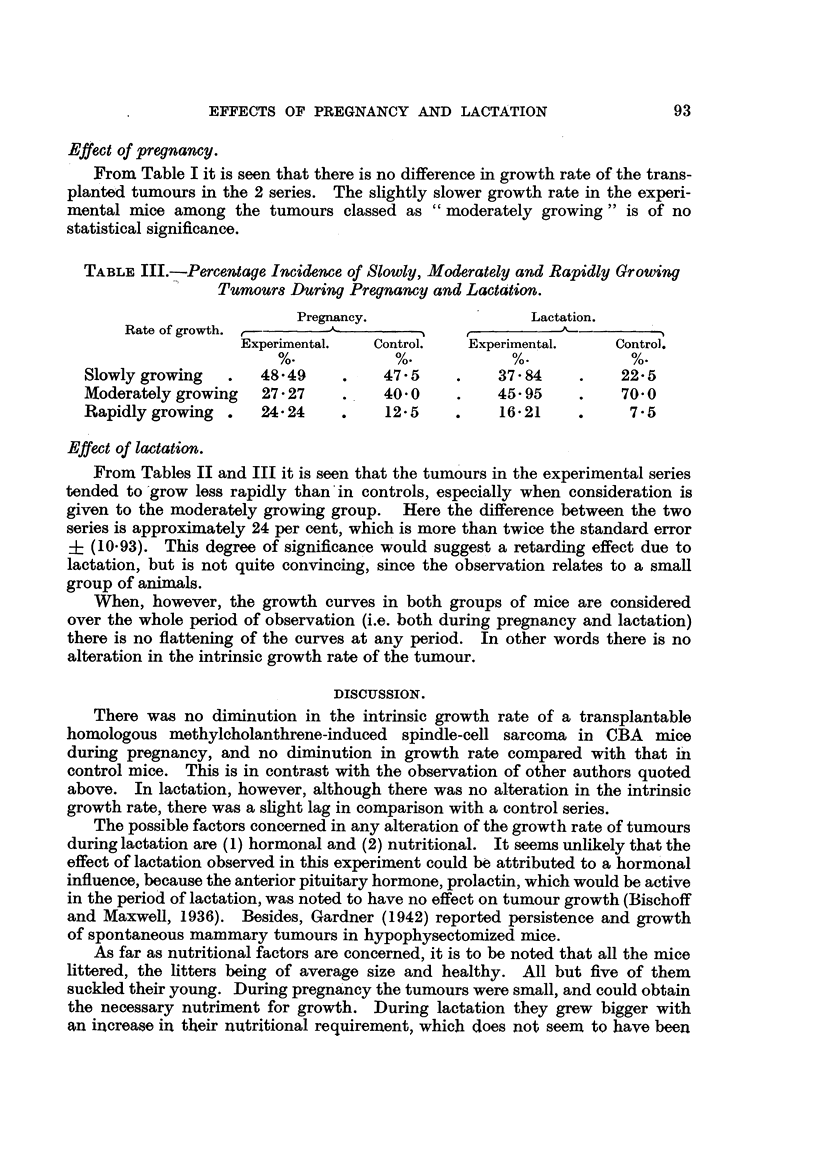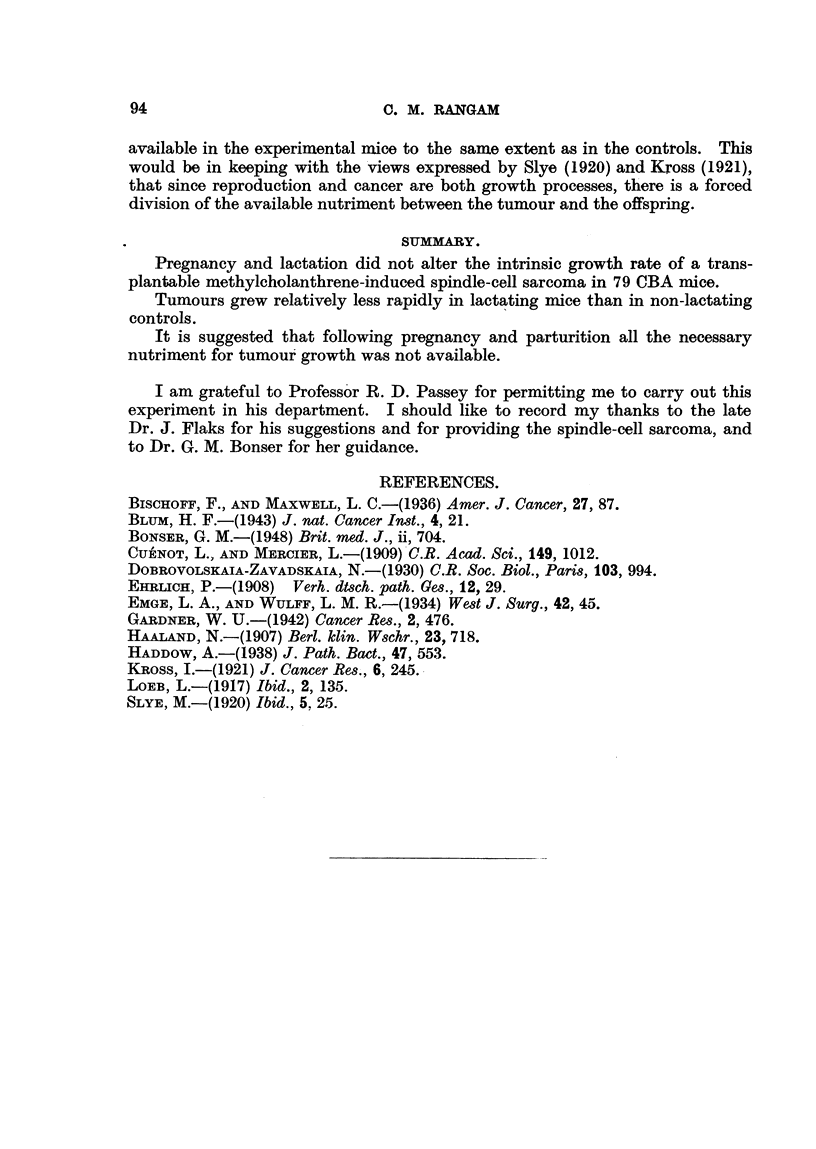# Effects of Pregnancy and Lactation on the Growth of a Transplantable Spindle Cell Sarcoma in CBA Mice

**DOI:** 10.1038/bjc.1950.7

**Published:** 1950-03

**Authors:** C. M. Rangam


### EFFECTS OF PREGNANCY AND LACTATION ON THE GROWTH OF A TRANSPLANTABLE SPINDLE CELL SARCOMA IN CBA MICE.

C. M. RANGAM.

*From the Department of Experimental Pathology and Cancer Research, University of Leeds.*

Received for publication January 17, 1950.

HAALAND (1907), Ehrlich (1908) and Loeb (1917) observed that in mice pregnancy was an unfavourable factor for the growth of transplanted tumours. Kross (1921) concluded that pregnancy had no effect on a transplantable rat carcinoma.

Haddow (1938) noted a decline in growth rate of spontaneous mammary carcinomas in mice during lactation, in the same way that Dobrovolskaia-Zavadskaia (1930) had reported partial regression of similar tumours during that period. Cuénot and Mercier (1909) recorded regression of Borrel's mouse tumour " B " during lactation, and Emge and Wulff (1934) found depression in growth curves of small fibromas and adenofibromas in the rat in the same period.

### MATERIAL AND METHODS.

In the present experiment sexually mature pure CBA female mice, bred in the laboratory and segregated from males for 2 months, were used. Three females and one young male mouse were kept together for 3 days in each box, during which time the females were examined every morning and evening for copulatory plugs. On the 4th day the males were removed from the boxes and 39 females which had shown plugs were placed in separate boxes, these constituting the experimental group. Forty-three unmated female mice of similar average age, and wherever possible litter mates of the experimental mice, made the control group.

On the day of separation, each of the mice received a subcutaneous graft, roughly 2 mm. $\times$ 2 mm., in the right flank, from a 25-day-old transplantable methylcholanthrene-induced spindle-cell sarcoma growing in CBA mice. This tumour retained the original histological appearance and characters of steady and uniform growth through successive generations, and was chosen because its rapid growth was considered an advantage in view of the shortness of the pregnancy and lactation periods in mice.

From the 10th day following grafting the tumours were measured every 4th or 5th day in 2 diameters (length and breadth) until the end of lactation, i.e. until 19 days following parturition. Two indices were calculated to assess the rates of growth of the tumours ; the index

$$\frac{a' \times b' \text{ (area on the day of parturition)}}{a \times b \text{ (area on the 10th day of grafting)}}$$ expresses the rate of growth during pregnancy and the index $\frac{a'' \times b'' \text{ (area at the end of lactation)}}{a' \times b' \text{ (area on the day of parturition)}}$ that during lactation. These indices have been accepted as proportional to the results obtained by using Blum's (1943) formula, assuming the shape of the tumour to be an ellipse (Bonser, 1948). The course of tumour growth is also represented in Fig. 1 by curves obtained by plotting the average of the sums of 2 diameters ($l + b$) against time, as described by Haddow (1938).

Because of the difficulty in obtaining a large number of pregnant mice at one time, the experiment had to be performed on 6 batches of mice, 25-day-old tumours of successive generations being used for grafting. When the tumours

FIG. 1.—Rate of tumour growth in experimental and control mice.
× ——— × Experimental.
⊗ - - - - ⊗ Control.

were not measured on the exact day of parturition or end of lactation, the measurements recorded on the day immediately preceding or following were employed. Four experimental and 2 control mice had tumours too small to measure on the 10th day of grafting, and their rates of growth during pregnancy could not be determined. Three mice (2 experimental and 1 control) grew more than one tumour close to one another. These are not included in the results. Five of the experimental mice killed their litters the day they were born. In all cases other CBA litters about a week old were substituted. The mice suckled them and the young grew normally.

### RESULTS.

In Tables I and II the growth rates of all tumours are recorded, and, employing an arbitrary classification into completely regressing, partially regressing, slowly growing, moderately growing, and rapidly growing tumours, arranged in five groups.

All but 2 of the tumours in the control mice grew steadily from their first appearance, and their course was represented by an approximately straight line (Fig. 1). One of the tumours regressed completely during the week following parturition, whilst the second began to regress during the same period and continued to regress to the end of the period of lactation.

TABLE I.—*Rate of Growth During Pregnancy* (*Summary of Indices*).

| $\frac{a' \times b'}{a \times b}$ | $\frac{a' \times b'}{a \times b}$ Experimental. | $\frac{a' \times b'}{a \times b}$ Control. | Rate of growth. |
|---|---|---|---|
| Disappeared | 0 | 0 | Complete regression. |
| <1 | 0 | 0 | Partial regression. |
| 1·0–1·99 | 6 | 9 | Slowly growing. |
| 2·0–2·99 | 10 | 10 | |
| 3·0–3·99 | 3 | 8 | Moderately growing. |
| 4·0–4·99 | 1 | 6 | |
| 5·0–5·99 | 5 | 2 | |
| 6·0–6·99 | 2 | 1 | Rapidly growing. |
| 7·0–7·99 | 4 | 1 | |
| >8 | 2 | 3 | |
| Total mice | 33 | 40 | |

TABLE II.—*Growth During Lacation* (*Summary of Indices*).

| $\frac{a'' \times b''}{a' \times b'}$ | $\frac{a'' \times b''}{a' \times b'}$ Experimental. | $\frac{a'' \times b''}{a' \times b'}$ Control. | Rate of growth. |
|---|---|---|---|
| Disappeared | 0 | 1 | Complete regression. |
| <1 | 0 | 1 | Partial regression. |
| 1·0–1·99 | 3 | 3 | Slowly growing. |
| 2·0–2·99 | 11 | 6 | |
| 3·0–3·99 | 6 | 13 | Moderately growing. |
| 4·0–4·99 | 7 | 11 | |
| 5·0–5·99 | 4 | 4 | |
| 6·0–6·99 | 5 | 2 | Rapidly growing. |
| 7·0–7·99 | 0 | 0 | |
| >8 | 1 | 1 | |
| Total mice | 37 | 42 | |

*Effect of pregnancy.*

From Table I it is seen that there is no difference in growth rate of the transplanted tumours in the 2 series. The slightly slower growth rate in the experimental mice among the tumours classed as "moderately growing" is of no statistical significance.

TABLE III.—*Percentage Incidence of Slowly, Moderately and Rapidly Growing Tumours During Pregnancy and Lactation.*

| Rate of growth. | Pregnancy. | | Lactation. | |
|---|---|---|---|---|
| | Experimental. % | Control. % | Experimental. % | Control. % |
| Slowly growing | 48·49 | 47·5 | 37·84 | 22·5 |
| Moderately growing | 27·27 | 40·0 | 45·95 | 70·0 |
| Rapidly growing | 24·24 | 12·5 | 16·21 | 7·5 |

*Effect of lactation.*

From Tables II and III it is seen that the tumours in the experimental series tended to grow less rapidly than in controls, especially when consideration is given to the moderately growing group. Here the difference between the two series is approximately 24 per cent, which is more than twice the standard error $\pm$ (10·93). This degree of significance would suggest a retarding effect due to lactation, but is not quite convincing, since the observation relates to a small group of animals.

When, however, the growth curves in both groups of mice are considered over the whole period of observation (i.e. both during pregnancy and lactation) there is no flattening of the curves at any period. In other words there is no alteration in the intrinsic growth rate of the tumour.

### DISCUSSION.

There was no diminution in the intrinsic growth rate of a transplantable homologous methylcholanthrene-induced spindle-cell sarcoma in CBA mice during pregnancy, and no diminution in growth rate compared with that in control mice. This is in contrast with the observation of other authors quoted above. In lactation, however, although there was no alteration in the intrinsic growth rate, there was a slight lag in comparison with a control series.

The possible factors concerned in any alteration of the growth rate of tumours during lactation are (1) hormonal and (2) nutritional. It seems unlikely that the effect of lactation observed in this experiment could be attributed to a hormonal influence, because the anterior pituitary hormone, prolactin, which would be active in the period of lactation, was noted to have no effect on tumour growth (Bischoff and Maxwell, 1936). Besides, Gardner (1942) reported persistence and growth of spontaneous mammary tumours in hypophysectomized mice.

As far as nutritional factors are concerned, it is to be noted that all the mice littered, the litters being of average size and healthy. All but five of them suckled their young. During pregnancy the tumours were small, and could obtain the necessary nutriment for growth. During lactation they grew bigger with an increase in their nutritional requirement, which does not seem to have been available in the experimental mice to the same extent as in the controls. This would be in keeping with the views expressed by Slye (1920) and Kross (1921), that since reproduction and cancer are both growth processes, there is a forced division of the available nutriment between the tumour and the offspring.

### SUMMARY.

Pregnancy and lactation did not alter the intrinsic growth rate of a transplantable methylcholanthrene-induced spindle-cell sarcoma in 79 CBA mice.

Tumours grew relatively less rapidly in lactating mice than in non-lactating controls.

It is suggested that following pregnancy and parturition all the necessary nutriment for tumour growth was not available.

I am grateful to Professor R. D. Passey for permitting me to carry out this experiment in his department. I should like to record my thanks to the late Dr. J. Flaks for his suggestions and for providing the spindle-cell sarcoma, and to Dr. G. M. Bonser for her guidance.

### REFERENCES.

Bischoff, F., and Maxwell, L. C.—(1936) *Amer. J. Cancer*, **27**, 87.
Blum, H. F.—(1943) *J. nat. Cancer Inst.*, **4**, 21.
Bonser, G. M.—(1948) *Brit. med. J.*, ii, 704.
Cuénot, L., and Mercier, L.—(1909) *C.R. Acad. Sci.*, **149**, 1012.
Dobrovolskaia-Zavadskaia, N.—(1930) *C.R. Soc. Biol., Paris*, **103**, 994.
Ehrlich, P.—(1908) *Verh. dtsch. path. Ges.*, **12**, 29.
Emge, L. A., and Wulff, L. M. R.—(1934) *West J. Surg.*, **42**, 45.
Gardner, W. U.—(1942) *Cancer Res.*, **2**, 476.
Haaland, N.—(1907) *Berl. klin. Wschr.*, **23**, 718.
Haddow, A.—(1938) *J. Path. Bact.*, **47**, 553.
Kross, I.—(1921) *J. Cancer Res.*, **6**, 245.
Loeb, L.—(1917) *Ibid.*, **2**, 135.
Slye, M.—(1920) *Ibid.*, **5**, 25.